# *C*_2_-isomer of [Pd(tfd)]_6_ [tfd is S_2_C_2_(CF_3_)_2_] as its benzene solvate: a new member of the small but growing class of homoleptic palladium(II) monodi­thio­lenes in the form of hexa­meric cubes

**DOI:** 10.1107/S2056989017007897

**Published:** 2017-06-02

**Authors:** Joshua Moscattini, Alan J. Lough, Ulrich Fekl

**Affiliations:** a Department of Chemical and Physical Sciences, University of Toronto Mississauga, 3359 Mississauga Rd, Mississauga, Ontario, L5L 1C6, Canada; b Department of Chemistry, University of Toronto, 80 St. George Street, Toronto, Ontario, M5S 3H6, Canada

**Keywords:** crystal structure, cube, hexa­meric cube, isomer, *C*_2_-symmetric

## Abstract

The title compound, hexa­kis­[μ_3_-1,2-bis­(tri­fluoro­meth­yl)ethene-1,2-di­thiol­ato]-*octa­hedro*-hexa­palladium(II), [Pd(C_4_F_6_S_2_)]_6_, crystallizes as its benzene solvate, [Pd(tfd)]_6_·2.5C_6_H_6_, where tfd is the di­thiol­ene S_2_C_2_(CF_3_)_2_. The mol­ecular structure of [Pd(tfd)]_6_ is of the hexa­metallic cube type seen previously in three examples of hexa­meric homoleptic palladium monodi­thiol­ene structures. All structures have in common: (*a*) the cluster closely approximates a cube containing six Pd^II^ atoms, one at the centre of each cube face; (*b*) 12 S atoms occupy the mid-points of all 12 cube edges, providing for each Pd^II^ atom an approximately square-planar *S*_4_ environment; (*c*) each S atom is part of a di­thiol­ene mol­ecule, where the size of the di­thiol­ene ligand necessitates that only sulfur atoms on adjacent cube edges can be part of the same di­thiol­ene. This general cube-type framework has so far given rise to two isomeric types: an *S*_6_-symmetric isomer and a *C*_2_-chiral type (two isomers that are enanti­omers of each other). The structure of [Pd(tfd)]_6_ is of the *C*_2_-type. Out of the 12 CF_3_ groups, three are rotationally disordered over two positions. Further, we answer the question of whether additional, previously undiscovered, isomers could follow from the cube rules (*a*) through (*c*) above. An exhaustive analysis shows that no additional isomers are possible and that the list of isomers (one *S*_6_ isomer, two *C*_2_ enanti­omers) is complete. Each isomer type could give rise to an unlimited number of compounds if the specific di­thiol­ene used is varied.

## Abstract 2

*S*_6_- and *C*_2_-symmetric structure types are possible for homoleptic palladium monodi­thiol­enes in the form of hexa­meric cubes. The *C*_2_-isomer of [Pd(S_2_C_2_(CF_3_)_2_)]_6_ is described here and the question of whether additional isomers are possible is answered.

## Chemical context   

Metal–di­thiol­ene complexes (di­thiol­ene = S_2_C_2_ *R*_2_) are relevant for a host of new materials and several metalloenzymes (Stiefel, 2004 ▸; Harrison *et al.*, 2006 ▸). Homoleptic complexes normally contain two or three di­thiol­enes per metal, for bis­dithiol­enes *M*(di­thiol­ene)_2_ or tris­dithiol­enes *M*(di­thiol­ene)_3_. *Monomeric* homoleptic monodi­thiol­enes of the composition *M*(di­thiol­ene)_1_ are unstable and polymerize or oligomerize. A *dimeric* complex [Ni(S_2_C_2_(CF_3_)_2_)]_2_ was recently computed (Dang *et al.*, 2013 ▸). The class of *hexa­meric* homoleptic palladium monodi­thiol­enes [Pd(di­thiol­ene)]_6_, where di­thiol­ene = *R*_2_C_2_S_2_ with any substituent *R* was suggested by Stiefel and co-workers (Beswick *et al.*, 2002 ▸), and a charge-neutral hexa­nuclear complex was crystallographically characterized (as its toluene solvate) using the di­thiol­ene S_2_C_2_(COOMe)_2_. The partially reduced complex, with a tetra­phenyl­phospho­nium counter-ion, was later structurally characterized by Stibrany (2012 ▸). A charge-neutral complex Pd(di­thiol­ene)_6_ with the di­thiol­ene S_2_C_6_H_2_(OMe)_2_ was reported by Rawson and co-workers (Wrixon *et al.*, 2015 ▸). In this work, we generated [Pd(tfd)]_6_ [where tfd is the di­thiol­ene S_2_C_2_(CF_3_)_2_] from tfd and the di­benzyl­ideneacetone (dba) complex Pd_2_dba_3_ as described below. A crystal was obtained, and the structure was determined by X-ray crystallography.
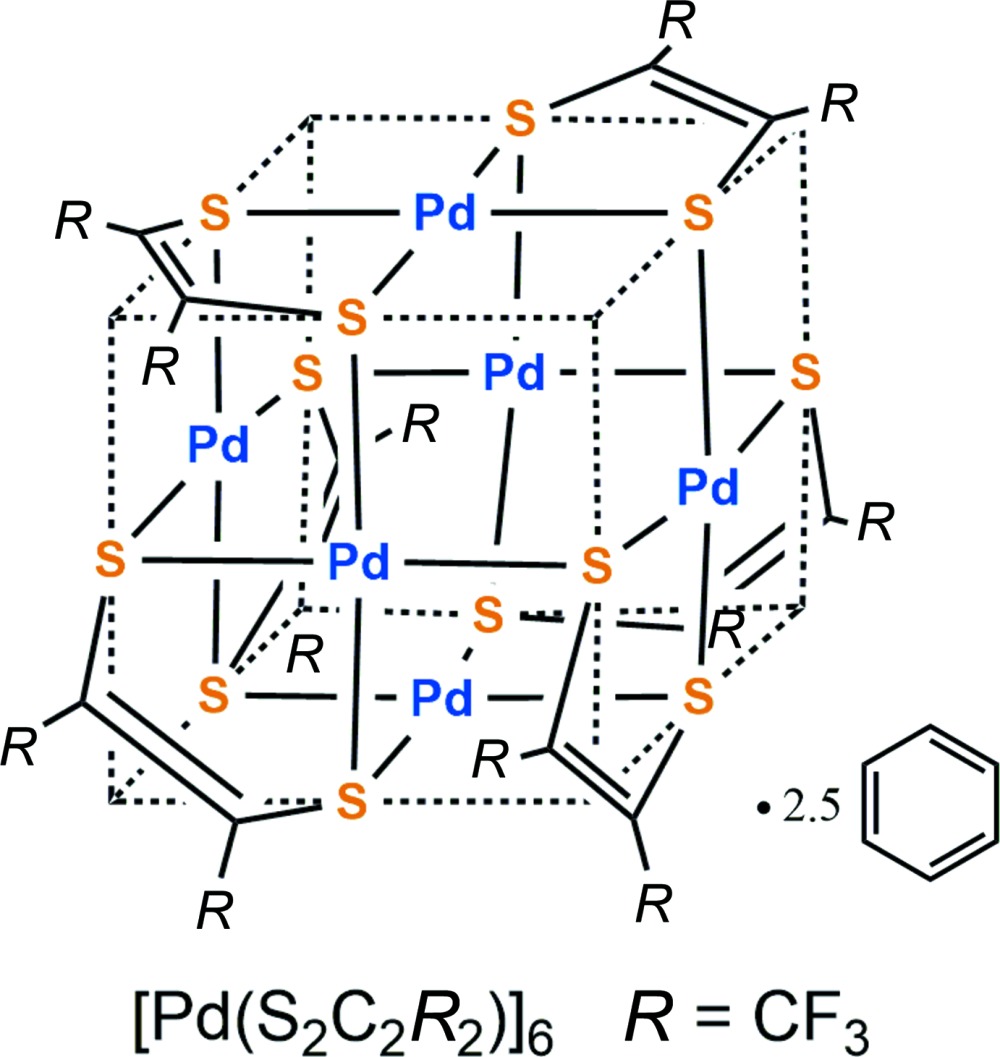


## Structural commentary   

**Figure 1:**
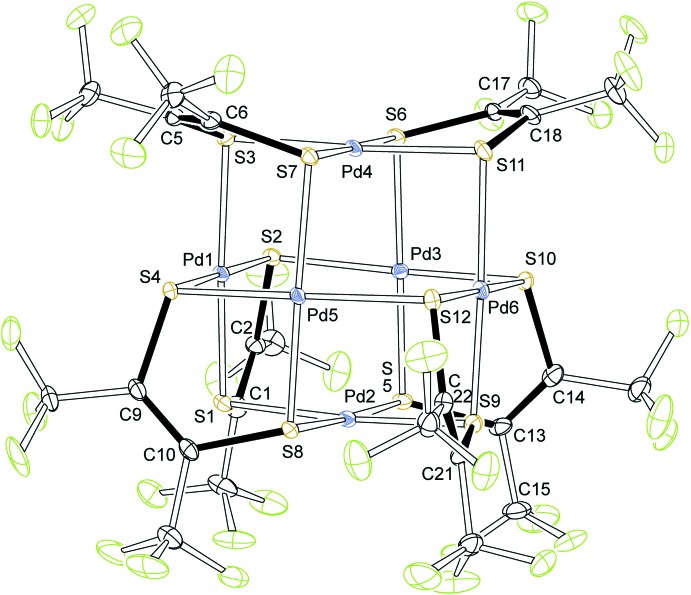
A view of the mol­ecular structure of [Pd(tfd)]_6_. Anisotropic displacement ellipsoids are shown at the 30% probability level. The ligand backbones are highlighted.

**Figure 2:**
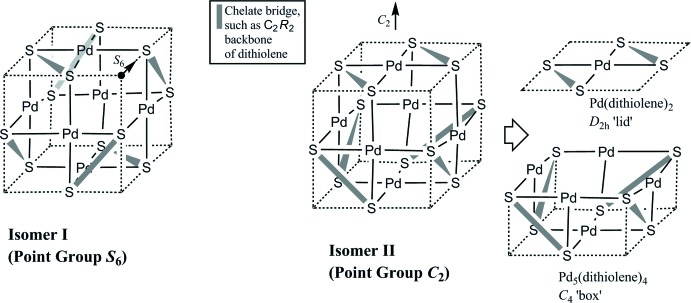
Isomers for [Pd(di­thiol­ene)]_6_.

The molecular structure of [Pd(tfd)]_6_ is shown in Fig. 1 ▸, where the second position for the rotationally disordered tri­fluoro­methyl groups (attached to C1, C13, and C14), the second position for disordered atom C15, as well as the benzene solvate mol­ecules are not displayed. The structure has approximate, non-crystallographic, *C*_2_ symmetry (*C*_2_ through Pd2 and Pd4). The gross features of this cube-like structure will be discussed first, followed by details such as bond lengths. The structure is of the hexa­metallic cube type seen previously in the hexa­meric homoleptic palladium monodi­thiol­enes characterized by Stiefel and co-workers (Beswick *et al.*, 2002 ▸), Stibrany (Stibrany, 2012 ▸), and Rawson and co-workers (Wrixon *et al.* 2015 ▸). All structures have in common: (*a*) the cluster closely approximates a cube containing six Pd^II^ atoms, one at the centre of each cube face; (*b*) 12 S atoms occupy the midpoint of all 12 cube edges, providing for each Pd^II^ atom an approximately square-planar *S*_4_ environment; (*c*) each S atom is part of a di­thiol­ene mol­ecule, where the size of the di­thiol­ene ligand requires that only sulfurs on adjacent cube edges can be part of the same di­thiol­ene. This general cube-type framework has so far given rise to two isomer types: one *S*_6_-symmetric isomer seen for the charge-neutral palladium complex of S_2_C_2_(COOMe)_2_ (Beswick *et al.*, 2002 ▸) and for a partially reduced complex involving the same ligand (Stibrany, 2012 ▸). The charge-neutral complex Pd(di­thiol­ene)_6_ with the di­thiol­ene S_2_C_6_H_2_(OMe)_2_ was found by Rawson and co-workers (Wrixon *et al.*, 2015 ▸) to have a different, *C*_2_-symmetric, structure. The two isomeric types are shown here schematically, inscribed into a cube (Fig. 2 ▸).

**Figure 3:**
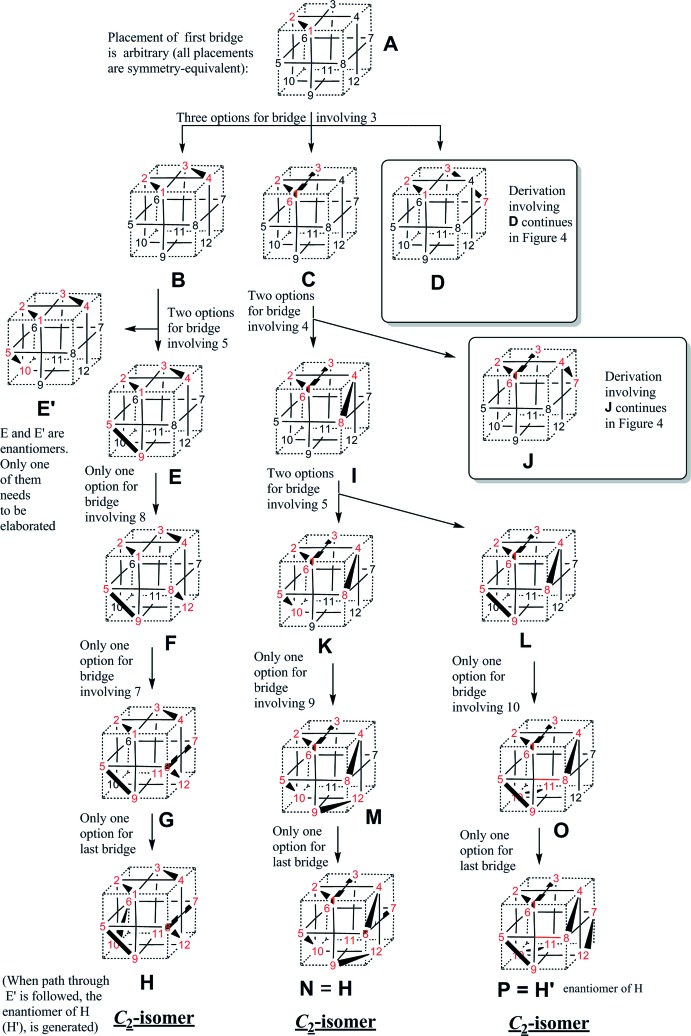
Graphical proof that only one *S*_6_ isomer and two (enanti­omeric) *C*_2_ isomers are possible for homoleptic palladium monodi­thiol­enes in the form of hexa­meric cubes, given the ‘cube rules’ discussed in the text. Continues in Fig. 4 ▸.

The question of whether additional isomers are possible remained open and is answered here (Figs. 3 ▸ and 4 ▸). The starting point is the constraint that 12 donor atoms (such as sulfur) reside at the midpoint of the 12 cube edges and that metal atoms (such as Pd) occupy the centres of all cube faces. The ligand length constraint forbids *trans*-spanning placement of a chelate bridge, and chelates can only bridge the short distance between adjacent edge positions. All possible isomers following from this framework are explored in an exhaustive fashion (Figs. 3 ▸ and 4 ▸), following the initially arbitrary placement of the first bridge. It results that no additional isomers are possible and that the list of isomers (one *S*_6_ isomer, two *C*_2_ enanti­omers) is complete. It is worthwhile noting that the *S*_6_ isomer cannot have a dipole moment, by virtue of its inversion centre, while the *C*_2_ isomer has a dipole.

With these general results for homoleptic square-planar metal-based short-span chelates in the fom of hexa­meric cubes [*M*(*L*_2_)]_6_ in hand, we return to discussing the details of the structure of [Pd(tfd)]_6_ (Fig. 1 ▸). It is notable that not all Pd—S distances are the same. Shorter distances, on average 2.294 Å (12 values; standard deviation = 0.008 Å), are found for the Pd—S distances within an approximately planar C_2_S_2_Pd five-membered ring. Longer distances, on average 2.364 Å (12 values; standard deviation = 0.01 Å) are found for coordination of an S atom outside its own C_2_S_2_Pd ring onto a different Pd^II^ atom at an approximately right angle to the five-membered ring. All bonds to Pd2, the Pd atom at the bottom of the Pd_5_(tfd)_4_ *C*_4_-symmetric ‘box’ (Fig. 2 ▸) are long. All bonds to Pd_4_, the Pd^II^ atom in the Pd(tfd)_2_ ‘lid’ are short. The charge on the tfd ligand can be seen from the C—C distance within the chelate ring, which is short (double bond) for the dianion (ene-di­thiol­ate; C—C distance of 1.35 Å or shorter expected) and long for monoanionic tfd (C—C bond order = 1.5; C—C distance of 1.38 Å expected), as is known from Tang *et al.* (2009 ▸) and Kogut *et al.* (2006 ▸) (see analysis in Hosking *et al.*, 2009 ▸). The chelate C—C bond distances in the current structure of [Pd(tfd)]_6_ average to 1.339 Å (six values; standard deviation = 0.006 Å) and indicate a dianionic chelate. Charge balance necessitates that all palladium atoms are in the oxidation state 2+, which is also supported by the coordination geometry around each Pd^II^ atom, which is approximately square-planar, as expected for a *d* ^8^ metal centre. The structure may thus be described as a charge-neutral *C*_4_-symmetric Pd_4_(di­thiol­ene)_4_ tiara capped on one side with a Pd^2+^ dication and on the other side with a Pd(tfd)_2_^2−^ dianion. While the structure is likely more charge balanced than this zwitterionic description implies, this description suggests a direction of the dipole moment.

## Supra­molecular features   

Mol­ecules of [Pd(tfd)]_6_ and benzene solvate mol­ecules pack *via* contacting van der Waals surfaces. There are no particularly short inter­molecular distances (such as hydrogen bonds).

## Database survey   

The three structures discussed (EGIDIH: Beswick *et al.*, 2002 ▸; XARMOU: Stibrany, 2012 ▸; YUQHUP: Wrixon *et al.*, 2015 ▸) are the only structures for hexa­metallic compounds of the type [Pd(di­thiol­ene)]_6_ in CSD (Groom *et al.*, 2016 ▸), Version 5.38 including updates up to Feb 2017.

## Synthesis and crystallization   

General specifications: All manipulations were carried out under an inert (N_2_) atmosphere using standard glove box (M. Braun UniLab) and Schlenk techniques. NMR solvents were obtained from Cambridge Isotope Laboratories. Solvents were purified prior to use by vacuum distillation from purple sodium benzo­phenone ketyl. NMR data were obtained on a Bruker Avance III 400 MHz spectrometer. Pd_2_dba_3_ was obtained from Sigma–Aldrich. S_2_C_2_(CF_3_)_2_ (tfd) was synthesized as in Harrison *et al.* (2006 ▸).

Synthesis: A pyrex reaction vessel containing 10 ml of toluene, 80 mg of Pd_2_dba_3_ (175 µmol of Pd) and 80 µl (350 µmol) of tfd was heated to 353 K overnight. At the end of the reaction, the dark-red solution had turned an intense brown. Solvent and volatiles were removed under vacuum at room temperature, followed by heating to 383 K for 4 h, also under vacuum. NMR spectroscopy (C_6_D_6_ solvent) showed a complex mixture, as indicated by multiple ^19^F resonances, chiefly two intense quartets (*J*_F–F_ = 14.5 Hz) at −58.7 ppm and −59.3 ppm but also a large number of overlapping signals between −56 and −58 ppm. *C*_2_-[Pd(tfd)]_6_ is clearly not the only species in solution, as it would give rise to six distinct fluorine signals (quartets) in equal intensity. It seems likely that multiple species are in equilibrium in the reaction mixture, and *C*_2_-[Pd(tfd)]_6_ might crystallize from non-polar solvents relatively early due to its dipole moment, which makes it less soluble in non-polar solvents compared to non-polar species. Dissolving the sample in a minimal amount of benzene, followed by storage at 285 K for one week, led to the formation of crystals suitable for X-ray crystallography.

## Refinement   

Crystal data, data collection and structure refinement details are summarized in Table 1 ▸. The F atoms of three of the –CF_3_ groups were refined as disordered over two sets of sites with the ratios of refined occupancies being 0.898 (6):0.102 (6) for F1/F2/F3, 0.784 (7):0.216 (7) for F19/F20/F21 and 0.749 (9):0.251 (9) for F22/F23/F24. Both the major and minor components were refined with anisotropic displacement parameters. In the –CF_3_ group containing F19/F20/F21, the attached atom C15 was also refined over two sets of sites with occupancies of 0.784 (7) and 0.216 (7). The SIMU command in *SHELXL* (Sheldrick, 2015 ▸) was used to restrain the anisotropic displacement parameters of the disordered atoms. The asymmetric unit contains 2.5 benzene solvent mol­ecules. One benzene mol­ecule is disordered about an inversion centre and hence has 0.5 occupancy. The RIGU command in *SHELXL* was used to restrain the anisotropic displacement parameters of the 0.5 occupancy benzene mol­ecule. The H atoms bonded to C atoms were placed in calculated positions C—H = 0.95 Å) and included in the refinement in a riding-motion approximation with *U*_iso_(H) = 1.2*U*_eq_(C).

## Supplementary Material

Crystal structure: contains datablock(s) I. DOI: 10.1107/S2056989017007897/zl2703sup1.cif

Structure factors: contains datablock(s) I. DOI: 10.1107/S2056989017007897/zl2703Isup2.hkl

CCDC reference: 1552666

Additional supporting information:  crystallographic information; 3D view; checkCIF report

## Figures and Tables

**Figure 4:**
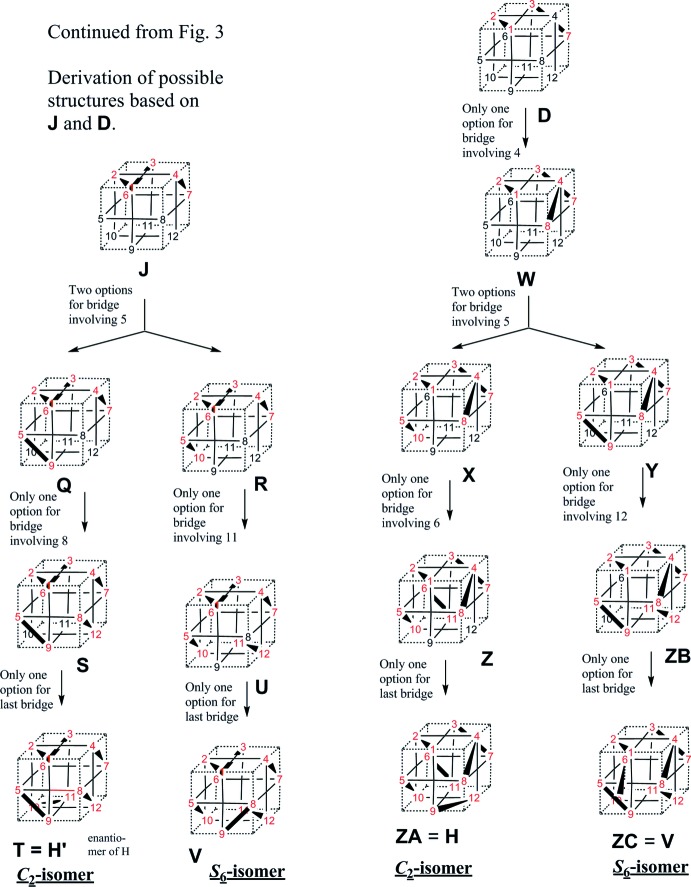
Continued from Fig. 3 ▸.

**Table 1:** Experimental details

Crystal data	
Chemical formula	[Pd_6_(C_4_F_6_S_2_)_6_]·2.5C_6_H_6_
*M*_r_	2190.63
Crystal system, space group	Monoclinic, *P*2_1_/*n*
Temperature (K)	147
*a*, *b*, *c* (Å)	15.6367 (15), 17.8970 (17), 22.532 (2)
β (°)	104.502 (2)
*V* (Å^3^)	6104.6 (10)
*Z*	4
Radiation type	Mo *K*α
μ (mm^−1^)	2.28
Crystal size (mm)	0.26 × 0.18 × 0.12

Data collection	
Diffractometer	Bruker Kappa *APEX* DUO CCD
Absorption correction	Multi-scan (*SADABS*; Bruker, 2014 ▸)
*T*_min_, *T*_max_	0.615, 0.746
No. of measured, independent and observed [*I* > 2σ(*I*)] reflections	52442, 13977, 10755
*R*_int_	0.035
(sin θ/λ)_max_ (Å^−1^)	0.651

Refinement	
*R*[*F* ^2^ > 2σ(*F* ^2^)], *wR*(*F* ^2^), *S*	0.040, 0.091, 1.11
No. of reflections	13977
No. of parameters	955
No. of restraints	390
H-atom treatment	H-atom parameters constrained
	*w* = 1/[σ^2^(*F*_o_^2^) + (0.016*P*)^2^ + 46.8497*P*] where *P* = (*F*_o_^2^ + 2*F*_c_^2^)/3
Δρ_max_, Δρ_min_ (e Å^−3^)	2.95, −1.37

Computer programs: *APEX2* and *SAINT* (Bruker, 2014 ▸), *SHELXS97* and *SHELXTL* (Sheldrick, 2008 ▸), *SHELXL2014* (Sheldrick, 2015 ▸) and *ORTEP-3 for Windows* (Farrugia, 2012 ▸).

## supplementary crystallographic information

### Crystal data

**Table:**

[Pd_6_(C_4_F_6_S_2_)_6_]·2.5C_6_H_6_	*F*(000) = 4164
*M**_r_* = 2190.63	*D*_x_ = 2.384 Mg m^−^^3^
Monoclinic, *P*2_1_/*n*	Mo *K*α radiation, λ = 0.71073 Å
*a* = 15.6367 (15) Å	Cell parameters from 9843 reflections
*b* = 17.8970 (17) Å	θ = 2.6–27.5°
*c* = 22.532 (2) Å	µ = 2.28 mm^−^^1^
β = 104.502 (2)°	*T* = 147 K
*V* = 6104.6 (10) Å^3^	Plate, bronze
*Z* = 4	0.26 × 0.18 × 0.12 mm

### Data collection

**Table:**

Bruker Kappa APEX DUO CCD diffractometer	10755 reflections with *I* > 2σ(*I*)
Radiation source: sealed tube with Bruker Triumph monochromator	*R*_int_ = 0.035
φ and ω scans	θ_max_ = 27.6°, θ_min_ = 1.4°
Absorption correction: multi-scan (SADABS; Bruker, 2014)	*h* = −20→20
*T*_min_ = 0.615, *T*_max_ = 0.746	*k* = −23→13
52442 measured reflections	*l* = −24→29
13977 independent reflections	

### Refinement

**Table:**

Refinement on *F*^2^	390 restraints
Least-squares matrix: full	Hydrogen site location: inferred from neighbouring sites
*R*[*F*^2^ > 2σ(*F*^2^)] = 0.040	H-atom parameters constrained
*wR*(*F*^2^) = 0.091	*w* = 1/[σ^2^(*F*_o_^2^) + (0.016*P*)^2^ + 46.8497*P*] where *P* = (*F*_o_^2^ + 2*F*_c_^2^)/3
*S* = 1.11	(Δ/σ)_max_ = 0.002
13977 reflections	Δρ_max_ = 2.95 e Å^−^^3^
955 parameters	Δρ_min_ = −1.37 e Å^−^^3^

### Special details

**Table:**

Geometry. All esds (except the esd in the dihedral angle between two l.s. planes) are estimated using the full covariance matrix. The cell esds are taken into account individually in the estimation of esds in distances, angles and torsion angles; correlations between esds in cell parameters are only used when they are defined by crystal symmetry. An approximate (isotropic) treatment of cell esds is used for estimating esds involving l.s. planes.

### Fractional atomic coordinates and isotropic or equivalent isotropic displacement parameters (Å^2^)

**Table:**

	*x*	*y*	*z*	*U*_iso_*/*U*_eq_	Occ. (<1)
Pd1	0.85082 (2)	0.76886 (2)	0.12870 (2)	0.01462 (8)	
Pd2	0.69610 (3)	0.86599 (2)	0.02884 (2)	0.01675 (9)	
Pd3	0.88930 (3)	0.84026 (2)	−0.00212 (2)	0.01563 (8)	
Pd4	0.86910 (3)	0.65611 (2)	0.01559 (2)	0.01621 (8)	
Pd5	0.67829 (2)	0.68175 (2)	0.04773 (2)	0.01439 (8)	
Pd6	0.71858 (3)	0.75118 (2)	−0.08355 (2)	0.01713 (9)	
S1	0.77482 (8)	0.87687 (7)	0.13250 (6)	0.0170 (2)	
S2	0.95772 (8)	0.84289 (7)	0.10438 (6)	0.0167 (2)	
S3	0.93246 (8)	0.65959 (7)	0.11965 (6)	0.0167 (2)	
S4	0.74009 (8)	0.69237 (7)	0.15081 (5)	0.0149 (2)	
S5	0.80145 (9)	0.94209 (7)	−0.00043 (6)	0.0195 (3)	
S6	0.97628 (8)	0.73296 (7)	−0.00334 (6)	0.0180 (3)	
S7	0.76397 (8)	0.57656 (7)	0.03498 (6)	0.0168 (2)	
S8	0.59617 (8)	0.78441 (7)	0.05793 (6)	0.0163 (2)	
S9	0.62407 (8)	0.84575 (7)	−0.07509 (6)	0.0192 (3)	
S10	0.82459 (8)	0.83395 (7)	−0.10505 (6)	0.0180 (3)	
S11	0.81170 (9)	0.64732 (7)	−0.08874 (6)	0.0202 (3)	
S12	0.61665 (9)	0.67151 (7)	−0.05945 (6)	0.0184 (3)	
F1	0.8470 (4)	1.0803 (2)	0.1229 (3)	0.0534 (15)	0.898 (6)
F2	0.8460 (5)	1.0370 (3)	0.2113 (2)	0.0607 (16)	0.898 (6)
F3	0.7328 (3)	1.0268 (2)	0.1361 (3)	0.0514 (14)	0.898 (6)
F1A	0.773 (3)	1.056 (2)	0.108 (2)	0.053 (6)	0.102 (6)
F2A	0.895 (3)	1.073 (2)	0.173 (2)	0.048 (6)	0.102 (6)
F3A	0.787 (3)	1.019 (2)	0.196 (2)	0.044 (6)	0.102 (6)
F4	1.0151 (3)	1.0393 (2)	0.1694 (2)	0.0555 (12)	
F5	1.0809 (3)	0.9587 (2)	0.1285 (2)	0.0561 (12)	
F6	0.9840 (3)	1.0317 (2)	0.07257 (19)	0.0524 (11)	
F7	0.9602 (3)	0.6236 (2)	0.24481 (16)	0.0412 (9)	
F8	0.9826 (3)	0.5113 (2)	0.21972 (18)	0.0456 (10)	
F9	0.8612 (3)	0.5410 (2)	0.24268 (16)	0.0400 (9)	
F10	0.8314 (3)	0.4306 (2)	0.16301 (18)	0.0415 (9)	
F11	0.7340 (3)	0.4355 (2)	0.07866 (18)	0.0469 (10)	
F12	0.7111 (3)	0.4900 (2)	0.1581 (2)	0.0499 (11)	
F13	0.6177 (3)	0.7686 (3)	0.26571 (18)	0.0595 (13)	
F14	0.7290 (4)	0.6985 (3)	0.27256 (17)	0.0662 (15)	
F15	0.7436 (3)	0.8158 (2)	0.26936 (16)	0.0475 (10)	
F16	0.4784 (3)	0.8280 (2)	0.1618 (2)	0.0564 (12)	
F17	0.5903 (3)	0.8950 (3)	0.2033 (2)	0.0617 (13)	
F18	0.5304 (3)	0.9068 (2)	0.10894 (19)	0.0540 (12)	
C13	0.7558 (9)	0.9641 (5)	−0.0788 (7)	0.0252 (15)	0.784 (7)
C15	0.7020 (6)	1.0358 (5)	−0.0888 (4)	0.0387 (18)	0.784 (7)
F19	0.7489 (4)	1.0925 (3)	−0.0989 (4)	0.066 (2)	0.784 (7)
F20	0.6305 (4)	1.0310 (3)	−0.1355 (3)	0.0591 (18)	0.784 (7)
F21	0.6704 (4)	1.0510 (3)	−0.0405 (3)	0.0520 (16)	0.784 (7)
C13A	0.757 (4)	0.9666 (17)	−0.079 (3)	0.032 (4)	0.216 (7)
C15A	0.7193 (15)	1.0451 (13)	−0.0853 (11)	0.041 (3)	0.216 (7)
F19A	0.7626 (14)	1.0918 (11)	−0.0445 (10)	0.065 (5)	0.216 (7)
F20A	0.7169 (16)	1.0759 (12)	−0.1394 (9)	0.057 (5)	0.216 (7)
F21A	0.6353 (13)	1.0436 (12)	−0.0823 (12)	0.061 (5)	0.216 (7)
F22	0.7870 (5)	0.8980 (4)	−0.2219 (2)	0.060 (2)	0.749 (9)
F23	0.6536 (4)	0.9084 (5)	−0.2135 (3)	0.058 (2)	0.749 (9)
F24	0.7377 (5)	1.0031 (4)	−0.2071 (3)	0.0483 (17)	0.749 (9)
F22A	0.7728 (14)	0.9736 (13)	−0.2162 (9)	0.049 (4)	0.251 (9)
F23A	0.7155 (15)	0.8665 (11)	−0.2199 (8)	0.056 (4)	0.251 (9)
F24A	0.6466 (13)	0.9614 (14)	−0.2020 (9)	0.059 (5)	0.251 (9)
F25	1.0181 (3)	0.7861 (2)	−0.15739 (17)	0.0396 (9)	
F26	1.0830 (3)	0.8072 (2)	−0.06401 (18)	0.0429 (10)	
F27	1.1060 (2)	0.7041 (2)	−0.10540 (18)	0.0407 (9)	
F28	0.9689 (3)	0.6493 (2)	−0.19661 (17)	0.0453 (10)	
F29	0.8409 (3)	0.6047 (2)	−0.20204 (18)	0.0525 (12)	
F30	0.8545 (3)	0.7203 (2)	−0.22092 (16)	0.0479 (11)	
F31	0.3878 (3)	0.8338 (3)	−0.0521 (2)	0.0592 (13)	
F32	0.4726 (3)	0.9211 (3)	−0.0621 (3)	0.0756 (17)	
F33	0.4036 (3)	0.8610 (3)	−0.1401 (2)	0.083 (2)	
F34	0.4510 (3)	0.6093 (2)	−0.0710 (2)	0.0506 (11)	
F35	0.4151 (3)	0.6926 (3)	−0.01466 (18)	0.0495 (11)	
F36	0.3690 (2)	0.6994 (2)	−0.11211 (17)	0.0460 (10)	
C1	0.8539 (4)	0.9489 (3)	0.1357 (3)	0.0231 (11)	
C2	0.9320 (4)	0.9340 (3)	0.1246 (2)	0.0196 (11)	
C3	0.8214 (4)	1.0246 (3)	0.1518 (3)	0.0348 (14)	
C4	1.0036 (4)	0.9916 (3)	0.1231 (3)	0.0315 (13)	
C5	0.8808 (4)	0.5831 (3)	0.1471 (2)	0.0198 (11)	
C6	0.8141 (4)	0.5462 (3)	0.1103 (2)	0.0191 (10)	
C7	0.9215 (4)	0.5637 (3)	0.2139 (3)	0.0280 (13)	
C8	0.7717 (4)	0.4751 (3)	0.1280 (3)	0.0279 (13)	
C9	0.6713 (4)	0.7557 (3)	0.1776 (2)	0.0209 (11)	
C10	0.6111 (4)	0.7969 (3)	0.1377 (2)	0.0212 (11)	
C11	0.6903 (4)	0.7603 (3)	0.2468 (2)	0.0270 (12)	
C12	0.5512 (4)	0.8566 (3)	0.1537 (3)	0.0309 (13)	
C14	0.7675 (4)	0.9193 (3)	−0.1231 (3)	0.0242 (12)	
C16	0.7327 (4)	0.9315 (4)	−0.1921 (3)	0.0346 (14)	
C17	0.9684 (4)	0.7189 (3)	−0.0817 (2)	0.0204 (11)	
C18	0.9005 (4)	0.6807 (3)	−0.1173 (3)	0.0254 (12)	
C19	1.0447 (4)	0.7546 (3)	−0.1028 (3)	0.0286 (13)	
C20	0.8913 (4)	0.6641 (4)	−0.1851 (3)	0.0333 (14)	
C21	0.5226 (3)	0.8006 (3)	−0.0753 (2)	0.0232 (11)	
C22	0.5192 (4)	0.7264 (3)	−0.0694 (2)	0.0229 (11)	
C23	0.4460 (4)	0.8544 (4)	−0.0831 (3)	0.0286 (13)	
C24	0.4372 (4)	0.6820 (4)	−0.0670 (3)	0.0292 (13)	
C1S	0.5486 (13)	0.3676 (7)	−0.1795 (6)	0.114 (5)	
H1SA	0.5377	0.3376	−0.2154	0.137*	
C2S	0.4802 (9)	0.3953 (6)	−0.1563 (5)	0.091 (4)	
H2SA	0.4206	0.3860	−0.1776	0.109*	
C3S	0.4977 (8)	0.4354 (6)	−0.1035 (5)	0.078 (3)	
H3SA	0.4501	0.4515	−0.0875	0.094*	
C4S	0.5816 (8)	0.4529 (6)	−0.0732 (5)	0.080 (3)	
H4SA	0.5910	0.4822	−0.0370	0.096*	
C5S	0.6526 (9)	0.4301 (8)	−0.0928 (6)	0.105 (4)	
H5SA	0.7107	0.4434	−0.0708	0.126*	
C6S	0.6388 (11)	0.3874 (9)	−0.1451 (7)	0.122 (5)	
H6SA	0.6879	0.3706	−0.1591	0.146*	
C7S	1.1256 (5)	0.8252 (5)	0.2648 (3)	0.051 (2)	
H7SA	1.1797	0.8088	0.2574	0.062*	
C8S	1.1131 (6)	0.8993 (5)	0.2753 (3)	0.054 (2)	
H8SA	1.1590	0.9342	0.2755	0.065*	
C9S	1.0345 (6)	0.9235 (5)	0.2855 (3)	0.055 (2)	
H9SA	1.0264	0.9748	0.2932	0.066*	
C10S	0.9668 (5)	0.8726 (5)	0.2844 (3)	0.0486 (19)	
H10A	0.9119	0.8892	0.2903	0.058*	
C11S	0.9801 (5)	0.7991 (4)	0.2749 (3)	0.0408 (16)	
H11A	0.9343	0.7642	0.2749	0.049*	
C12S	1.0593 (5)	0.7743 (4)	0.2652 (3)	0.0438 (17)	
H12A	1.0681	0.7227	0.2588	0.053*	
C13S	1.0775 (8)	0.5324 (8)	0.0644 (5)	0.071 (4)	0.5
H13A	1.1182	0.5488	0.1009	0.085*	0.5
C14S	1.0094 (9)	0.4841 (7)	0.0679 (5)	0.063 (4)	0.5
H14A	1.0034	0.4674	0.1067	0.076*	0.5
C15S	0.9499 (7)	0.4602 (7)	0.0146 (7)	0.058 (4)	0.5
H15A	0.9033	0.4271	0.0169	0.070*	0.5
C16S	0.9586 (8)	0.4845 (8)	−0.0422 (5)	0.075 (4)	0.5
H16A	0.9179	0.4682	−0.0786	0.089*	0.5
C17S	1.0267 (9)	0.5329 (8)	−0.0457 (5)	0.058 (4)	0.5
H17A	1.0327	0.5496	−0.0845	0.069*	0.5
C18S	1.0862 (7)	0.5568 (7)	0.0077 (7)	0.074 (5)	0.5
H18A	1.1328	0.5899	0.0053	0.088*	0.5

### Atomic displacement parameters (Å^2^)

**Table:**

	*U*^11^	*U*^22^	*U*^33^	*U*^12^	*U*^13^	*U*^23^
Pd1	0.01603 (19)	0.01422 (18)	0.01529 (18)	0.00025 (14)	0.00707 (15)	0.00090 (13)
Pd2	0.01734 (19)	0.01621 (19)	0.01881 (19)	0.00263 (14)	0.00848 (16)	0.00203 (14)
Pd3	0.01624 (19)	0.01545 (19)	0.01734 (19)	0.00239 (14)	0.00818 (15)	0.00321 (14)
Pd4	0.0200 (2)	0.01513 (19)	0.01698 (19)	0.00253 (14)	0.01104 (16)	0.00223 (14)
Pd5	0.01651 (19)	0.01516 (18)	0.01306 (18)	0.00024 (14)	0.00665 (15)	0.00022 (13)
Pd6	0.0181 (2)	0.0204 (2)	0.01470 (18)	0.00038 (15)	0.00743 (15)	0.00177 (14)
S1	0.0196 (6)	0.0151 (6)	0.0187 (6)	0.0000 (5)	0.0089 (5)	−0.0018 (4)
S2	0.0147 (6)	0.0179 (6)	0.0191 (6)	−0.0007 (4)	0.0071 (5)	0.0006 (5)
S3	0.0162 (6)	0.0168 (6)	0.0190 (6)	0.0027 (5)	0.0076 (5)	0.0035 (5)
S4	0.0164 (6)	0.0169 (6)	0.0127 (5)	0.0006 (4)	0.0061 (5)	0.0013 (4)
S5	0.0206 (6)	0.0153 (6)	0.0252 (7)	0.0033 (5)	0.0106 (5)	0.0037 (5)
S6	0.0189 (6)	0.0177 (6)	0.0209 (6)	0.0030 (5)	0.0113 (5)	0.0035 (5)
S7	0.0213 (6)	0.0136 (6)	0.0181 (6)	−0.0001 (5)	0.0097 (5)	−0.0001 (4)
S8	0.0165 (6)	0.0183 (6)	0.0160 (6)	0.0018 (5)	0.0073 (5)	0.0008 (4)
S9	0.0161 (6)	0.0246 (7)	0.0185 (6)	0.0021 (5)	0.0074 (5)	0.0057 (5)
S10	0.0179 (6)	0.0220 (7)	0.0165 (6)	0.0028 (5)	0.0087 (5)	0.0063 (5)
S11	0.0268 (7)	0.0202 (7)	0.0176 (6)	−0.0001 (5)	0.0128 (5)	−0.0017 (5)
S12	0.0191 (6)	0.0221 (7)	0.0147 (6)	−0.0032 (5)	0.0055 (5)	−0.0004 (5)
F1	0.065 (3)	0.019 (2)	0.093 (4)	0.000 (2)	0.052 (3)	0.006 (2)
F2	0.094 (5)	0.046 (3)	0.042 (3)	0.019 (3)	0.017 (3)	−0.023 (2)
F3	0.038 (3)	0.023 (2)	0.098 (4)	0.0080 (18)	0.027 (3)	−0.008 (2)
F1A	0.064 (11)	0.025 (10)	0.077 (11)	0.013 (10)	0.031 (11)	−0.014 (10)
F2A	0.064 (11)	0.026 (10)	0.064 (11)	0.005 (10)	0.034 (10)	−0.013 (10)
F3A	0.056 (11)	0.024 (10)	0.060 (11)	−0.001 (10)	0.030 (10)	−0.018 (10)
F4	0.067 (3)	0.046 (3)	0.055 (3)	−0.034 (2)	0.017 (2)	−0.015 (2)
F5	0.032 (2)	0.040 (2)	0.101 (4)	−0.0098 (18)	0.025 (2)	0.000 (2)
F6	0.058 (3)	0.051 (3)	0.048 (2)	−0.028 (2)	0.013 (2)	0.017 (2)
F7	0.055 (3)	0.039 (2)	0.0246 (18)	−0.0074 (18)	0.0003 (17)	0.0047 (15)
F8	0.046 (2)	0.043 (2)	0.043 (2)	0.0202 (18)	0.0028 (19)	0.0157 (18)
F9	0.051 (2)	0.046 (2)	0.0264 (19)	−0.0060 (18)	0.0172 (18)	0.0131 (16)
F10	0.050 (2)	0.0226 (19)	0.053 (2)	−0.0005 (16)	0.014 (2)	0.0164 (16)
F11	0.062 (3)	0.030 (2)	0.046 (2)	−0.0234 (19)	0.010 (2)	0.0023 (17)
F12	0.053 (3)	0.040 (2)	0.071 (3)	−0.0028 (19)	0.045 (2)	0.013 (2)
F13	0.045 (3)	0.115 (4)	0.029 (2)	−0.005 (2)	0.029 (2)	−0.009 (2)
F14	0.127 (5)	0.052 (3)	0.020 (2)	0.030 (3)	0.020 (2)	0.0067 (18)
F15	0.054 (3)	0.062 (3)	0.0247 (19)	−0.020 (2)	0.0075 (18)	−0.0139 (18)
F16	0.035 (2)	0.054 (3)	0.096 (4)	0.0072 (19)	0.045 (2)	0.001 (2)
F17	0.060 (3)	0.063 (3)	0.061 (3)	0.019 (2)	0.012 (2)	−0.037 (2)
F18	0.075 (3)	0.043 (2)	0.056 (3)	0.033 (2)	0.040 (2)	0.014 (2)
C13	0.027 (3)	0.021 (3)	0.033 (3)	0.009 (3)	0.017 (3)	0.015 (3)
C15	0.044 (4)	0.030 (4)	0.048 (4)	0.021 (3)	0.021 (3)	0.014 (3)
F19	0.073 (4)	0.027 (3)	0.105 (6)	0.012 (3)	0.037 (4)	0.027 (4)
F20	0.051 (3)	0.064 (4)	0.057 (4)	0.039 (3)	0.004 (3)	0.016 (3)
F21	0.068 (4)	0.048 (3)	0.046 (3)	0.041 (3)	0.026 (3)	0.012 (3)
C13A	0.035 (6)	0.026 (6)	0.039 (6)	0.013 (6)	0.018 (6)	0.016 (6)
C15A	0.046 (5)	0.033 (5)	0.049 (5)	0.018 (5)	0.018 (5)	0.018 (5)
F19A	0.075 (9)	0.036 (8)	0.074 (9)	0.028 (7)	−0.001 (8)	0.014 (8)
F20A	0.076 (9)	0.038 (8)	0.055 (8)	0.020 (7)	0.012 (8)	0.033 (7)
F21A	0.055 (8)	0.056 (8)	0.069 (9)	0.024 (7)	0.014 (8)	0.023 (8)
F22	0.072 (5)	0.083 (5)	0.028 (3)	0.040 (4)	0.020 (3)	0.016 (3)
F23	0.040 (3)	0.088 (6)	0.036 (3)	−0.025 (4)	−0.009 (3)	0.022 (3)
F24	0.067 (4)	0.040 (4)	0.037 (3)	0.010 (3)	0.013 (3)	0.028 (3)
F22A	0.047 (9)	0.057 (9)	0.041 (8)	−0.013 (7)	0.005 (7)	0.031 (7)
F23A	0.075 (10)	0.055 (8)	0.032 (7)	0.009 (8)	0.001 (7)	0.014 (7)
F24A	0.046 (8)	0.074 (10)	0.048 (8)	−0.001 (8)	−0.004 (7)	0.021 (8)
F25	0.048 (2)	0.042 (2)	0.039 (2)	0.0030 (17)	0.0293 (19)	0.0157 (17)
F26	0.050 (2)	0.040 (2)	0.049 (2)	−0.0169 (18)	0.030 (2)	−0.0025 (18)
F27	0.034 (2)	0.043 (2)	0.055 (2)	0.0116 (16)	0.0312 (19)	0.0087 (18)
F28	0.051 (2)	0.057 (3)	0.040 (2)	0.0063 (19)	0.034 (2)	−0.0085 (18)
F29	0.070 (3)	0.062 (3)	0.036 (2)	−0.023 (2)	0.031 (2)	−0.0237 (19)
F30	0.062 (3)	0.062 (3)	0.0216 (19)	0.017 (2)	0.0141 (19)	0.0102 (17)
F31	0.044 (3)	0.071 (3)	0.077 (3)	0.018 (2)	0.041 (2)	0.016 (2)
F32	0.034 (2)	0.043 (3)	0.147 (5)	0.012 (2)	0.018 (3)	−0.009 (3)
F33	0.073 (3)	0.139 (5)	0.034 (2)	0.076 (4)	0.005 (2)	0.021 (3)
F34	0.033 (2)	0.037 (2)	0.084 (3)	−0.0099 (17)	0.017 (2)	0.007 (2)
F35	0.041 (2)	0.076 (3)	0.037 (2)	−0.021 (2)	0.0197 (19)	−0.004 (2)
F36	0.0226 (19)	0.074 (3)	0.035 (2)	−0.0152 (18)	−0.0040 (16)	0.0093 (19)
C1	0.023 (3)	0.019 (3)	0.027 (3)	−0.005 (2)	0.007 (2)	−0.002 (2)
C2	0.022 (3)	0.015 (3)	0.021 (3)	−0.001 (2)	0.005 (2)	−0.0029 (19)
C3	0.041 (3)	0.023 (3)	0.049 (4)	−0.001 (2)	0.027 (3)	−0.008 (3)
C4	0.032 (3)	0.023 (3)	0.041 (4)	−0.011 (2)	0.011 (3)	−0.002 (3)
C5	0.027 (3)	0.015 (3)	0.020 (3)	0.006 (2)	0.011 (2)	0.0058 (19)
C6	0.025 (3)	0.012 (2)	0.024 (3)	0.0019 (19)	0.011 (2)	0.0034 (19)
C7	0.034 (3)	0.023 (3)	0.025 (3)	0.000 (2)	0.004 (3)	0.008 (2)
C8	0.031 (3)	0.024 (3)	0.032 (3)	−0.004 (2)	0.014 (3)	0.009 (2)
C9	0.028 (3)	0.021 (3)	0.017 (3)	−0.001 (2)	0.012 (2)	−0.001 (2)
C10	0.023 (3)	0.024 (3)	0.021 (3)	−0.003 (2)	0.014 (2)	−0.001 (2)
C11	0.035 (3)	0.033 (3)	0.015 (3)	−0.001 (2)	0.011 (2)	−0.002 (2)
C12	0.033 (3)	0.033 (3)	0.032 (3)	0.002 (3)	0.017 (3)	−0.004 (3)
C14	0.019 (3)	0.028 (3)	0.027 (3)	0.000 (2)	0.008 (2)	0.012 (2)
C16	0.033 (3)	0.042 (4)	0.030 (3)	0.008 (3)	0.009 (3)	0.016 (3)
C17	0.023 (3)	0.022 (3)	0.019 (3)	0.006 (2)	0.013 (2)	0.005 (2)
C18	0.031 (3)	0.028 (3)	0.024 (3)	0.007 (2)	0.019 (3)	0.001 (2)
C19	0.037 (3)	0.025 (3)	0.031 (3)	0.004 (2)	0.021 (3)	0.004 (2)
C20	0.043 (4)	0.038 (4)	0.024 (3)	−0.002 (3)	0.018 (3)	−0.007 (3)
C21	0.014 (3)	0.036 (3)	0.018 (3)	0.006 (2)	0.000 (2)	0.000 (2)
C22	0.025 (3)	0.027 (3)	0.016 (3)	0.002 (2)	0.003 (2)	0.000 (2)
C23	0.019 (3)	0.036 (4)	0.031 (3)	0.005 (2)	0.006 (2)	0.007 (2)
C24	0.024 (3)	0.039 (4)	0.025 (3)	−0.001 (2)	0.008 (3)	0.005 (2)
C1S	0.204 (18)	0.071 (8)	0.070 (8)	−0.025 (10)	0.041 (10)	−0.021 (6)
C2S	0.122 (10)	0.058 (7)	0.083 (8)	−0.049 (7)	0.005 (7)	0.011 (6)
C3S	0.086 (8)	0.070 (7)	0.081 (7)	−0.022 (6)	0.025 (6)	0.014 (6)
C4S	0.088 (8)	0.079 (7)	0.068 (7)	−0.021 (6)	0.012 (6)	0.010 (5)
C5S	0.089 (9)	0.137 (12)	0.085 (9)	0.005 (8)	0.015 (7)	−0.013 (8)
C6S	0.133 (13)	0.124 (13)	0.125 (13)	0.025 (10)	0.064 (11)	−0.003 (10)
C7S	0.040 (4)	0.077 (6)	0.035 (4)	−0.002 (4)	0.004 (3)	−0.002 (4)
C8S	0.058 (5)	0.070 (6)	0.032 (4)	−0.017 (4)	0.007 (4)	0.007 (4)
C9S	0.081 (6)	0.045 (5)	0.034 (4)	0.006 (4)	0.004 (4)	0.001 (3)
C10S	0.052 (5)	0.063 (5)	0.031 (4)	0.010 (4)	0.010 (3)	0.002 (3)
C11S	0.041 (4)	0.052 (5)	0.026 (3)	−0.003 (3)	0.002 (3)	0.000 (3)
C12S	0.048 (4)	0.048 (4)	0.032 (4)	−0.001 (3)	0.004 (3)	−0.005 (3)
C13S	0.070 (7)	0.060 (7)	0.082 (7)	0.032 (5)	0.016 (5)	−0.014 (5)
C14S	0.065 (7)	0.059 (7)	0.068 (7)	0.028 (5)	0.020 (5)	−0.007 (5)
C15S	0.061 (7)	0.041 (6)	0.071 (6)	0.032 (5)	0.016 (5)	−0.010 (5)
C16S	0.075 (7)	0.068 (7)	0.081 (7)	0.030 (6)	0.019 (5)	−0.007 (5)
C17S	0.061 (6)	0.050 (6)	0.064 (7)	0.038 (5)	0.019 (5)	−0.002 (5)
C18S	0.069 (7)	0.067 (8)	0.085 (7)	0.028 (6)	0.019 (5)	−0.005 (5)

### Geometric parameters (Å, º)

**Table:**

Pd1—S1	2.2819 (13)	F22—C16	1.349 (8)
Pd1—S2	2.3040 (13)	F23—C16	1.279 (9)
Pd1—S4	2.3564 (12)	F24—C16	1.333 (9)
Pd1—S3	2.3718 (13)	F22A—C16	1.195 (18)
Pd1—Pd5	3.2516 (6)	F23A—C16	1.32 (2)
Pd2—S8	2.3486 (13)	F24A—C16	1.41 (2)
Pd2—S5	2.3554 (13)	F25—C19	1.321 (7)
Pd2—S9	2.3586 (14)	F26—C19	1.322 (7)
Pd2—S1	2.3599 (13)	F27—C19	1.331 (7)
Pd3—S5	2.2883 (13)	F28—C20	1.331 (7)
Pd3—S10	2.2892 (13)	F29—C20	1.322 (7)
Pd3—S6	2.3574 (13)	F30—C20	1.328 (8)
Pd3—S2	2.3706 (13)	F31—C23	1.330 (7)
Pd3—Pd6	3.2528 (6)	F32—C23	1.312 (8)
Pd4—S6	2.2892 (13)	F33—C23	1.294 (7)
Pd4—S7	2.2979 (13)	F34—C24	1.327 (7)
Pd4—S11	2.3021 (14)	F35—C24	1.323 (7)
Pd4—S3	2.3065 (13)	F36—C24	1.313 (7)
Pd5—S8	2.2858 (13)	C1—C2	1.333 (7)
Pd5—S4	2.2892 (13)	C1—C3	1.522 (8)
Pd5—S7	2.3695 (13)	C2—C4	1.528 (7)
Pd5—S12	2.3725 (13)	C5—C6	1.334 (8)
Pd6—S9	2.2860 (13)	C5—C7	1.521 (7)
Pd6—S12	2.3026 (13)	C6—C8	1.532 (7)
Pd6—S10	2.3620 (13)	C9—C10	1.347 (8)
Pd6—S11	2.3822 (14)	C9—C11	1.515 (7)
S1—C1	1.775 (5)	C10—C12	1.523 (7)
S2—C2	1.767 (5)	C14—C16	1.529 (8)
S3—C5	1.778 (5)	C17—C18	1.346 (8)
S4—C9	1.770 (5)	C17—C19	1.530 (8)
S5—C13	1.774 (15)	C18—C20	1.527 (8)
S5—C13A	1.78 (6)	C21—C22	1.339 (8)
S6—C17	1.757 (5)	C21—C23	1.513 (7)
S7—C6	1.768 (5)	C22—C24	1.519 (8)
S8—C10	1.768 (5)	C1S—C2S	1.395 (18)
S9—C21	1.779 (6)	C1S—C6S	1.47 (2)
S10—C14	1.765 (6)	C1S—H1SA	0.9500
S11—C18	1.774 (6)	C2S—C3S	1.356 (15)
S12—C22	1.779 (6)	C2S—H2SA	0.9500
F1—C3	1.307 (8)	C3S—C4S	1.356 (14)
F2—C3	1.317 (8)	C3S—H3SA	0.9500
F3—C3	1.343 (8)	C4S—C5S	1.355 (16)
F1A—C3	1.23 (5)	C4S—H4SA	0.9500
F2A—C3	1.43 (4)	C5S—C6S	1.375 (18)
F3A—C3	1.25 (4)	C5S—H5SA	0.9500
F4—C4	1.324 (7)	C6S—H6SA	0.9500
F5—C4	1.323 (7)	C7S—C8S	1.370 (12)
F6—C4	1.316 (7)	C7S—C12S	1.380 (11)
F7—C7	1.338 (7)	C7S—H7SA	0.9500
F8—C7	1.321 (7)	C8S—C9S	1.376 (12)
F9—C7	1.334 (7)	C8S—H8SA	0.9500
F10—C8	1.326 (7)	C9S—C10S	1.390 (12)
F11—C8	1.327 (7)	C9S—H9SA	0.9500
F12—C8	1.323 (7)	C10S—C11S	1.358 (10)
F13—C11	1.317 (7)	C10S—H10A	0.9500
F14—C11	1.323 (7)	C11S—C12S	1.384 (10)
F15—C11	1.314 (7)	C11S—H11A	0.9500
F16—C12	1.303 (7)	C12S—H12A	0.9500
F17—C12	1.324 (7)	C13S—C14S	1.3900
F18—C12	1.328 (7)	C13S—C18S	1.3900
C13—C14	1.328 (15)	C13S—H13A	0.9500
C13—C15	1.519 (9)	C14S—C15S	1.3900
C15—F19	1.306 (10)	C14S—H14A	0.9500
C15—F21	1.331 (9)	C15S—C16S	1.3900
C15—F20	1.334 (10)	C15S—H15A	0.9500
C13A—C14	1.35 (5)	C16S—C17S	1.3900
C13A—C15A	1.518 (16)	C16S—H16A	0.9500
C15A—F19A	1.301 (17)	C17S—C18S	1.3900
C15A—F20A	1.330 (17)	C17S—H17A	0.9500
C15A—F21A	1.331 (16)	C18S—H18A	0.9500
			
S1—Pd1—S2	86.42 (5)	F8—C7—F9	108.5 (5)
S1—Pd1—S4	93.84 (5)	F8—C7—F7	107.3 (5)
S2—Pd1—S4	178.49 (5)	F9—C7—F7	105.9 (5)
S1—Pd1—S3	176.57 (5)	F8—C7—C5	111.9 (5)
S2—Pd1—S3	90.89 (5)	F9—C7—C5	112.2 (5)
S4—Pd1—S3	88.80 (4)	F7—C7—C5	110.7 (4)
S1—Pd1—Pd5	93.47 (4)	F12—C8—F10	107.8 (5)
S2—Pd1—Pd5	133.76 (4)	F12—C8—F11	108.1 (5)
S4—Pd1—Pd5	44.74 (3)	F10—C8—F11	106.0 (5)
S3—Pd1—Pd5	86.86 (3)	F12—C8—C6	112.3 (5)
S8—Pd2—S5	176.83 (5)	F10—C8—C6	111.6 (5)
S8—Pd2—S9	89.77 (5)	F11—C8—C6	110.8 (5)
S5—Pd2—S9	90.25 (5)	C10—C9—C11	125.5 (5)
S8—Pd2—S1	89.64 (4)	C10—C9—S4	120.4 (4)
S5—Pd2—S1	90.08 (5)	C11—C9—S4	114.0 (4)
S9—Pd2—S1	175.18 (5)	C9—C10—C12	126.5 (5)
S5—Pd3—S10	86.63 (5)	C9—C10—S8	120.1 (4)
S5—Pd3—S6	178.23 (5)	C12—C10—S8	113.4 (4)
S10—Pd3—S6	93.32 (5)	F15—C11—F13	107.7 (5)
S5—Pd3—S2	95.05 (5)	F15—C11—F14	106.6 (5)
S10—Pd3—S2	178.23 (5)	F13—C11—F14	106.9 (5)
S6—Pd3—S2	84.98 (5)	F15—C11—C9	112.4 (5)
S5—Pd3—Pd6	89.34 (4)	F13—C11—C9	112.1 (5)
S10—Pd3—Pd6	46.55 (3)	F14—C11—C9	110.8 (5)
S6—Pd3—Pd6	89.35 (4)	F16—C12—F17	108.7 (5)
S2—Pd3—Pd6	132.79 (3)	F16—C12—F18	108.4 (5)
S6—Pd4—S7	178.58 (5)	F17—C12—F18	105.3 (5)
S6—Pd4—S11	88.12 (5)	F16—C12—C10	111.5 (5)
S7—Pd4—S11	92.14 (5)	F17—C12—C10	112.5 (5)
S6—Pd4—S3	91.43 (5)	F18—C12—C10	110.1 (4)
S7—Pd4—S3	88.23 (5)	C13—C14—C16	126.7 (7)
S11—Pd4—S3	176.66 (5)	C13A—C14—C16	125.9 (18)
S8—Pd5—S4	86.35 (4)	C13—C14—S10	120.4 (6)
S8—Pd5—S7	178.63 (5)	C13A—C14—S10	121.3 (17)
S4—Pd5—S7	94.66 (5)	C16—C14—S10	112.8 (4)
S8—Pd5—S12	93.96 (5)	F22A—C16—F23A	114.1 (15)
S4—Pd5—S12	178.97 (5)	F23—C16—F24	109.2 (6)
S7—Pd5—S12	85.01 (5)	F23—C16—F22	109.8 (7)
S8—Pd5—Pd1	88.07 (3)	F24—C16—F22	102.5 (6)
S4—Pd5—Pd1	46.43 (3)	F22A—C16—F24A	106.1 (14)
S7—Pd5—Pd1	91.96 (3)	F23A—C16—F24A	100.7 (14)
S12—Pd5—Pd1	132.58 (3)	F22A—C16—C14	117.4 (11)
S9—Pd6—S12	86.49 (5)	F23—C16—C14	113.7 (5)
S9—Pd6—S10	93.11 (5)	F23A—C16—C14	109.6 (8)
S12—Pd6—S10	178.24 (5)	F24—C16—C14	111.5 (6)
S9—Pd6—S11	176.11 (5)	F22—C16—C14	109.5 (5)
S12—Pd6—S11	89.78 (5)	F24A—C16—C14	107.3 (9)
S10—Pd6—S11	90.59 (5)	C18—C17—C19	125.9 (5)
S9—Pd6—Pd3	92.67 (4)	C18—C17—S6	121.1 (4)
S12—Pd6—Pd3	133.57 (4)	C19—C17—S6	112.9 (4)
S10—Pd6—Pd3	44.72 (3)	C17—C18—C20	123.9 (5)
S11—Pd6—Pd3	89.13 (4)	C17—C18—S11	121.9 (4)
C1—S1—Pd1	104.64 (19)	C20—C18—S11	114.2 (4)
C1—S1—Pd2	106.59 (19)	F25—C19—F26	107.3 (5)
Pd1—S1—Pd2	92.32 (4)	F25—C19—F27	108.2 (5)
C2—S2—Pd1	104.11 (18)	F26—C19—F27	107.4 (5)
C2—S2—Pd3	101.83 (18)	F25—C19—C17	112.3 (5)
Pd1—S2—Pd3	93.38 (5)	F26—C19—C17	110.5 (4)
C5—S3—Pd4	102.48 (18)	F27—C19—C17	110.9 (5)
C5—S3—Pd1	107.66 (17)	F29—C20—F30	107.3 (6)
Pd4—S3—Pd1	90.36 (4)	F29—C20—F28	106.9 (5)
C9—S4—Pd5	105.15 (18)	F30—C20—F28	108.0 (5)
C9—S4—Pd1	104.00 (18)	F29—C20—C18	110.3 (5)
Pd5—S4—Pd1	88.83 (4)	F30—C20—C18	112.3 (5)
C13—S5—Pd3	104.7 (3)	F28—C20—C18	111.9 (5)
C13A—S5—Pd3	105.4 (11)	C22—C21—C23	126.4 (5)
C13—S5—Pd2	105.7 (5)	C22—C21—S9	120.5 (4)
C13A—S5—Pd2	107.2 (19)	C23—C21—S9	113.1 (4)
Pd3—S5—Pd2	90.51 (5)	C21—C22—C24	125.2 (5)
C17—S6—Pd4	103.37 (19)	C21—C22—S12	120.4 (4)
C17—S6—Pd3	103.45 (18)	C24—C22—S12	114.3 (4)
Pd4—S6—Pd3	92.05 (5)	F33—C23—F32	108.3 (6)
C6—S7—Pd4	101.93 (18)	F33—C23—F31	107.4 (5)
C6—S7—Pd5	104.86 (17)	F32—C23—F31	105.0 (5)
Pd4—S7—Pd5	89.08 (4)	F33—C23—C21	111.4 (5)
C10—S8—Pd5	105.62 (18)	F32—C23—C21	111.5 (5)
C10—S8—Pd2	106.13 (18)	F31—C23—C21	112.9 (5)
Pd5—S8—Pd2	92.40 (5)	F36—C24—F35	108.3 (5)
C21—S9—Pd6	104.98 (19)	F36—C24—F34	107.1 (5)
C21—S9—Pd2	106.06 (18)	F35—C24—F34	106.4 (5)
Pd6—S9—Pd2	92.21 (5)	F36—C24—C22	112.2 (5)
C14—S10—Pd3	105.1 (2)	F35—C24—C22	111.6 (5)
C14—S10—Pd6	104.44 (18)	F34—C24—C22	110.9 (5)
Pd3—S10—Pd6	88.73 (4)	C2S—C1S—C6S	116.1 (11)
C18—S11—Pd4	101.9 (2)	C2S—C1S—H1SA	122.0
C18—S11—Pd6	107.36 (19)	C6S—C1S—H1SA	122.0
Pd4—S11—Pd6	89.04 (4)	C3S—C2S—C1S	120.8 (12)
C22—S12—Pd6	104.49 (19)	C3S—C2S—H2SA	119.6
C22—S12—Pd5	101.71 (18)	C1S—C2S—H2SA	119.6
Pd6—S12—Pd5	93.64 (5)	C4S—C3S—C2S	121.4 (12)
C14—C13—C15	125.0 (11)	C4S—C3S—H3SA	119.3
C14—C13—S5	121.1 (6)	C2S—C3S—H3SA	119.3
C15—C13—S5	113.9 (9)	C5S—C4S—C3S	122.3 (12)
F19—C15—F21	108.9 (8)	C5S—C4S—H4SA	118.9
F19—C15—F20	107.9 (8)	C3S—C4S—H4SA	118.9
F21—C15—F20	104.3 (7)	C4S—C5S—C6S	118.7 (13)
F19—C15—C13	111.7 (8)	C4S—C5S—H5SA	120.6
F21—C15—C13	110.9 (8)	C6S—C5S—H5SA	120.6
F20—C15—C13	112.8 (8)	C5S—C6S—C1S	120.6 (13)
C14—C13A—C15A	129 (4)	C5S—C6S—H6SA	119.7
C14—C13A—S5	119.4 (16)	C1S—C6S—H6SA	119.7
C15A—C13A—S5	112 (3)	C8S—C7S—C12S	119.9 (8)
F19A—C15A—F20A	105.7 (19)	C8S—C7S—H7SA	120.1
F19A—C15A—F21A	109 (2)	C12S—C7S—H7SA	120.1
F20A—C15A—F21A	104.6 (19)	C7S—C8S—C9S	120.3 (8)
F19A—C15A—C13A	114 (2)	C7S—C8S—H8SA	119.8
F20A—C15A—C13A	113 (2)	C9S—C8S—H8SA	119.8
F21A—C15A—C13A	110 (2)	C8S—C9S—C10S	119.9 (8)
C2—C1—C3	126.7 (5)	C8S—C9S—H9SA	120.0
C2—C1—S1	120.6 (4)	C10S—C9S—H9SA	120.0
C3—C1—S1	112.7 (4)	C11S—C10S—C9S	119.5 (8)
C1—C2—C4	125.4 (5)	C11S—C10S—H10A	120.3
C1—C2—S2	120.7 (4)	C9S—C10S—H10A	120.3
C4—C2—S2	113.7 (4)	C10S—C11S—C12S	120.8 (7)
F1A—C3—F3A	112 (3)	C10S—C11S—H11A	119.6
F1—C3—F2	109.3 (6)	C12S—C11S—H11A	119.6
F1—C3—F3	105.7 (6)	C7S—C12S—C11S	119.5 (7)
F2—C3—F3	106.4 (6)	C7S—C12S—H12A	120.2
F1A—C3—F2A	106 (3)	C11S—C12S—H12A	120.2
F3A—C3—F2A	105 (3)	C14S—C13S—C18S	120.0
F1A—C3—C1	113.3 (18)	C14S—C13S—H13A	120.0
F3A—C3—C1	110.8 (18)	C18S—C13S—H13A	120.0
F1—C3—C1	113.5 (5)	C15S—C14S—C13S	120.0
F2—C3—C1	111.3 (6)	C15S—C14S—H14A	120.0
F3—C3—C1	110.3 (5)	C13S—C14S—H14A	120.0
F2A—C3—C1	109.3 (16)	C14S—C15S—C16S	120.0
F6—C4—F5	109.3 (5)	C14S—C15S—H15A	120.0
F6—C4—F4	106.6 (5)	C16S—C15S—H15A	120.0
F5—C4—F4	105.9 (5)	C15S—C16S—C17S	120.0
F6—C4—C2	111.9 (5)	C15S—C16S—H16A	120.0
F5—C4—C2	110.9 (5)	C17S—C16S—H16A	120.0
F4—C4—C2	112.0 (5)	C16S—C17S—C18S	120.0
C6—C5—C7	124.8 (5)	C16S—C17S—H17A	120.0
C6—C5—S3	121.2 (4)	C18S—C17S—H17A	120.0
C7—C5—S3	114.0 (4)	C17S—C18S—C13S	120.0
C5—C6—C8	124.8 (5)	C17S—C18S—H18A	120.0
C5—C6—S7	122.3 (4)	C13S—C18S—H18A	120.0
C8—C6—S7	112.9 (4)		
